# SPDEF ameliorates UUO-induced renal fibrosis by transcriptional activation of NR4A1

**DOI:** 10.1186/s10020-024-01030-3

**Published:** 2024-12-30

**Authors:** Hongshuang Wang, Ziheng Wei, Chang Xu, Fang Fang, Zheng Wang, Yan Zhong, Xiangting Wang

**Affiliations:** 1 Hebei University of Chinese Medicine, Shijiazhuang, 050091 China; 2 The First Affiliated Hospital of Hebei University of Chinese Medicine, Shijiazhuang, 050011 China; 3 Hebei Key Laboratory of Integrated Chinese and Western Medicine for Lung Disease Research, Shijiazhuang, 050091 China; 4 Hebei Key Laboratory of Integrative Medicine On Liver-Kidney Patterns, Shijiazhuang, 050091 China; 5 Institute of Integrative Medicine, College of Integrative Medicine, Hebei University of Chinese Medicine, Shijiazhuang, 050200 China

**Keywords:** Chronic kidney disease, NR4A1, SPDEF, Renal fibrosis

## Abstract

Nuclear receptor 4A1 (NR4A1) is a gene that increases the likelihood of chronic kidney disease (CKD) and contributes to its development. Previous research has shown that the SAM pointed domain containing Ets transformation-specific transcription factor (SPDEF) can activate NR4A1, but its mechanism of action in renal fibrosis is not yet clear. In this study, we used adenovirus to create a mouse kidney model with a specific knockdown of NR4A1 gene. Our results showed that the knockdown of NR4A1 can accelerate unilateral ureteral obstruction (UUO)-induced renal fibrosis in mice, and overexpression of NR4A1 can significantly reduce transforming growth factor-β1-induced (TGF-β1) fibrosis in HK-2 cells. Additionally, we found that overexpression of SPDEF can improve UUO-induced renal fibrosis in mice and TGF-β1-induced fibrosis in HK-2 by transcriptionally activating NR4A1. These findings suggest that SPDEF can activate NR4A1 transcriptionally and improve renal fibrosis.

## Introduction

As a common clinical disease, chronic kidney disease (CKD) is an important factor leading to end-stage kidney disease (GBD Chronic Kidney Disease Collaboration 2017). Concealed onset and progression are the most significant clinical characteristics of CKD (Luyckx et al. 2020). The course of CKD is usually over for several years when the patients are diagnosed with CKD. Therefore, it is difficult to treat CKD with long-lasting therapy (Obrador and Levin 2019). Renal fibrosis is a common pathological pathway for various CKD to progress to end-stage renal failure (Humphreys 2018). The mechanism of renal fibrosis is currently unclear. Accordingly, it is crucial to explore its underlying mechanism for clinical research.

Nuclear receptor 4A1 (NR4A1) is a member of the nuclear receptor superfamily and can participate in the occurrence and development of CKD by regulating various pathways such as inflammation, oxidative stress, and mitochondrial function (Hedrick and Safe 2017; Westbrook et al. 2014). Our previous research has shown that NR4A1 expression is reduced in animal models of unilateral ureteral obstruction (UUO) induced renal fibrosis (He et al. 2022; Xu et al. 2023; Wang et al. 2024). Consequently, we speculate that increasing the expression of NR4A1 can effectively improve renal fibrosis.

The SAM pointed domain containing Ets transcription factor (SPDEF) is a member of the Ets transcription factor family, located on the P-arm of chromosome 6 (6p21.31) and involved in the encoding of 335 amino acids (aa) (Vatanmakanian et al. 2023). All Ets transcription factors share a highly conserved Ets DNA binding domain containing 85 aa. Previous studies have shown that SPDEF suppresses head and neck squamous cell carcinoma progression by transcriptionally activating NR4A1 (Wang et al. 2021). Currently, the role of SPDEF in CKD is unclear. We hypothesized that SPDEF could inhibit renal fibrosis by activating NR4A1.

We conducted experiments on UUO animal model and cellular fibrosis induced by transforming growth factor-β1 (TGF-β1). We studied the role of SPDEF and NR4A1 in renal fibrosis and their interaction by knocking down either of them. Our findings suggest that SPDEF can improve renal fibrosis by activating NR4A1 transcriptionally.

## Methods

### Animals, chemicals and antibodies

C57BL/6 J mice were purchased from Beijing Weitong Lihua Experimental Animal Technology Co., Ltd. (license number: SCXK (Beijing) 2016-0006), which were housed and maintained in a 12 h light/12 h dark cycle in a clean animal facility at the Hebei University of Chinese Medicine. Approved by the Animal Ethics Committee of Hebei University of Chinese Medicine (Approval No. DWLL202203129).

### Chemicals and antibodies see Table 1

**Table 1:** Antibody Reagent List

REAGENT or RESOURCE	SOURCE	IDENTIFIER
Antibodies		
α-SMA	Abcam	Cat: #ab5694
Vimentin	Abcam	Cat#: ab92547
Collagen I	Proteintech Abcam	Cat#:67288–1-lg Cat#:ab270993
NR4A1	Immunoway Proteintech	Cat#:YT3213 Cat#:12235–1-AP
GAPDH	Proteintech	Cat#:10494–1-AP
SPDEF	Proteintech	Cat#:11467–1-AP
Chemicals, peptides, and recombinant proteins		
DMEM	Gibco	Cat#:12491,015
Fetal Bovine Serum	Gibco	Cat#:10099
Penicillin and streptomycin	Gibco	Cat#:15070063
Recombinant Human TGF-β1	Proteintech	Cat#HZ-1011
Adenovirus: Ad-NR4A1	Hanheng	AP22112703
Adenovirus: Ad-con	Hanheng	AP21072502
Adenovirus: sh-NR4A1	Hanheng	AD21062310
Adenovirus: sh-con	Hanheng	AP21070906
Adenovirus: Ad-NR4A1	GenePharma	No.230624AZ
Adenovirus: Ad-con	GenePharma	No.E12ZZ
Adenovirus: sh-NR4A1	Genechem	Cat#:127058–1
Adenovirus: sh-con	Genechem	Cat#:LVCON313
Trypsin‑EDTA solution	Gibco	Cat#:25,200,072
Total RNA Kit II	Omega	Cat:#R6934-01
2*SYBR Green qPCR Master Mix kit	Servicebio	Cat#:G3326-05
Swescript All-in-one First-Strand cDNA Synthesis Super Mix for qPCR	Servicebio	Cat#:G3337-50
HRP-conjugated Affinipure Goat Anti-Rabbit gG (H + L)	Proteintech	Cat#:SA00001-2
HRP-conjugated Affinipure Goat Anti-Mouse gG (H + L)	Proteintech	Cat#:SA00001-1
Mayer, Hematoxylin solution	Solarbio	Cat#:G1080
Histochemistry kit	ZSGB-BIO	Cat#:2221c0725
Normal Goat Serum	Solarbio	Cat#:NO.SL038
DAB	ORIGENE	Cat#:220,030,819
Sodium Citrate buffer	Solarbio	Cat#:C1010
Nonfat dry milk	Biosharp	Cat#:NO.114A0164
Immobilon-E	Millipore	Cat#:R1CB73921
PH8.8	Solarbio	Cat#:T1010
PH6.8	Solarbio	Cat#:T1020
ACR	Solarbio	Cat#:CR2209071
TEMED	Solarbio	Cat#:T8090
NcmECL High	NCM Biotech	Cat NO:P10300
CoraLite488-conjugated Goat Anti-Mouse IgG (H + L)	Proteintech	Cat#:No.SA00013-1
CoraLite594 – conjugated Goat Anti-Rabbit IgG (H + L)	Proteintech	Cat#:No.SA00013-4

#### Establishment of UUO model

The method of model preparation is consistent with the previous method (He et al. 2022). Model started 1 week after adaptive feeding. After isoflurane inhalation anesthesia, the mice were placed on the operating table. The abdomen of the mice was exposed in the supine position. The right abdomen of the mouse was cut open, the kidney was left free, and ligation began after the ureter was found. After ligating the upper 1/3 and middle 1/3 of the ureter, respectively, the ureter between the two ligated ends was cut and the skin was sutured layer by layer. In sham group, the left ureter was exposed but not ligated. On the second day of mold making, 10 mg·kg^−1^·d^−1^ of Cytosporone B (dissolved in DMSO) was administered, and equal volume of saline was administered in the Sham and UUO groups. After waking up, place the animal in the animal room.

#### Intrarenal injections

The method of model preparation is consistent with the previous method (Wang et al. 2024). The lentiviral vectors were delivered into the kidneys of experimental mice by intrarenal injection. SPDEF knockdown mice method is the same as NR4A1 knockdown mice. Target sequence see Table 2.

**Table 2:** Target Seq

Species	Gene	Sit	Target Seq
Human	NR4A1	sh-NR4A1-1	CAGCTGCCAGGAACAGTCCAGCCAT
Human	NR4A1	sh-NR4A1-2	CAGCATATGGTGTCCGCACATGIG
Human	NR4A1	sh-NR4A1-3	CCACCCATCATTGACAAGATCTTCA
Human	SPDEF	sh-SPDEF-1	ACCTCTCCTACTTTGACATGC
Human	SPDEF	sh-SPDEF-2	GCAGTTCCTCAAGGAGTTGCT
Human	SPDEF	sh-SPDEF-3	GCTCCATCCGCCAGTATTACA
Mice	NR4A1	sh-NR4A1-1	GCCCTGTATTCAAGCTCAATA
Mice	NR4A1	sh-NR4A1-2	GCTTCGGCGTCCTTCAAGTTT
Mice	NR4A1	sh-NR4A1-3	GCCAGACTTATGAAGGCCTCT
Mice	SPDEF	sh-SPDEF-1	GCTTTCTACCTCTCTTACTTT
Mice	SPDEF	sh-SPDEF-2	GGCGAGGTCCTGAAAGATATT
Mice	SPDEF	sh-SPDEF-3	GCGCCTTGTCTACCAATTTGT

#### HE, masson, and sirius red

After fixing the tissue with paraformaldehyde, the tissue is dehydrated and dewaxed according to a gradient, followed by hematoxylin staining. After neutralization with hydrochloric acid, the tissue is rinsed under water for 1 h, and then dehydrated with alcohol. The tissue is stained with eosin for 2–3 min, dehydrated and transparent, and then sealed. HE staining (inflammatory cell infiltration and tubulointerstitial changes) were semi-quantitatively graded by two investigators. The two items were scored as 0 (minor), 1 (minor), 2 (moderate), and 3 (severe), with a total score ranging from 0 to 6. A semiquantitative analysis of Masson and Sirius red staining was performed according to the percentage of the collagen positive areas. Image analyses were performed using Image J 6.0 software (US National Institutes of Health, Bethesda, MD, USA). Muscle fibers appear red with Masson staining, while collagen fibers appear blue. Collagen fibers appear red or bright red with Sirius red staining, while others appear yellow.

#### Cell culture and treatment

Human proximal renal tubular epithelial cells (HK-2) were cultured in DMED containing 10% fetal bovine serum (FBS), 1% penicillin, and streptomycin, and maintained in a humidified incubator with 5% CO_2_ at 37 °C. We use TGF-β1 (10 ng/mL) to stimulate HK-2 for 24 h.

#### Lentiviral transfection

Before transfection, the cells were inoculated into a 6 cm cell culture vessel. The density of the cells during transfection was between 50% and 60% confluency. The cells were infected with the supernatant of the media containing the adenovirus. Next, 2 µl of the adenovirus plus the cells were infected twice using a 10^8^ TU/mL titer as well as transfection with Ad-con (negative control), sh-con (negative control), Ad-NR4A1 or sh-NR4A1. Finally, the transfection efficiency was verified and characterized. TGF-β1 was added to stimulate HK2 cells for 24 h after transfection. The recombinant adenovirus vector carrying sh-SPDEF or Ad-SPDEF was purchased from Genechem Co., Ltd. The transfection method was the same as sh-NR4A1 or Ad-NR4A1. Or simultaneously adding Ad-SPDEF and sh-NR4A1. Extract protein or RNA and perform relevant testing.

#### Western blot

After weighing kidney tissue, add lysate and protease inhibitors in proportion, cut the tissue, homogenize, centrifuge (with a radius of 8 cm), take the supernatant, add protein sample buffer, and denature for 10 min. Prepare 10% SDS-PAGE gel, sample with 20 µg protein, electrophoresis for 2 h, membrane transfer for 30 min, milk sealing for 2 h, and add a first antibody (SPDEF 1:500, NR4A1 1:500, α-SMA 1:1000, Vimentin 1:500, Collagen I 1:500). 4 °C being sealed overnight, TBST was cleaned and incubated at room temperature for 2 antibodies before scanning the membrane. The membrane was swept after four washes in TBST. Simultaneously GAPDH (1:3000) is used as an internal parameter correction. Use Image J for statistical analysis.

#### Immunohistochemistry (IHC)

Paraffin embedding, slicing, dewaxing, alcohol gradient dehydration, antigen repair, room temperature cooling, PBS cleaning, 3% hydrogen peroxide to block endogenous peroxidase, PBS cleaning, and addition of an antibody α-SMA (1:200), Vimentin (1:250), incubated overnight at 4 °C; Clean with PBS, incubated with secondary antibody at 37 °C for 1 h, clean with PBS, add horseradish peroxidase dropwise for 15 min, color with DAB, stain with hematoxylin, acidify with hydrochloric acid, clean with PBS, and seal with dehydration. Micrographs were photographed and analyzed by Image J.Brown indicates positive expression.

#### Immunofluorescence (IF)

After embedding the tissue with OCT, a frozen slicer was used to cut it to a thickness of 6 μm. After PBS cleaning, antigen repair was performed. After natural rewarming to room temperature, goat serum staining (37 °C, 20 min) was performed, and the first antibody was added (α-SMA 1:300 and Vimentin 1:300) Incubate overnight at 4 °C. On the second day, clean the first antibody and incubate the corresponding second antibody. After staining with DAPI, seal the film and observe the staining under a microscope.

#### ChIP-qPCR

After the cells were stimulated, paraformaldehyde was added, 30 µl per 1 ml of solution, making a final concentration of 1.42% formaldehyde, and the bed was shaken at room temperature for 10 min. Crosslinking was terminated by adding 115 µl of 1.25 mol/L glycine per 1 ml of solution, making a final concentration of 125 mM. Shaking the bed for 5 min at room temperature. After harvesting the cells, 1 ml of IP buffer was added, and the cells were resuspended after centrifugation and discarding the supernatant for 2–3 repetitions. Sonication was used to lyse the cells:50% of the output power, 15 pulses of 1 s at 2 min intervals, and repeated 3 times. Antibodies were added and shaken at 4 °C overnight. The next day add Beads, wash and shake on ice for 2–3 h. Centrifuge to remove supernatant and resuspend with 1 ml IP buffer. Add 50 µl double-distilled water, mediate for 10 s, and boil for 10 min. Centrifuge the supernatant, measure the DNA concentration, then perform real-time PCR or − 80 °C freezing and storage.

#### qRT-PCR

Total RNA was isolated from the tissue samples using RNA extraction and cDNA templates. Using a 2*SYBR Green qPCR Master Mix kit we performed real-time fluorescence quantitative polymerase chain reaction. ABI 7500 FAST system was used to detect the mRNA levels of related indicators. All primers were provided by Servicebio and Biotech. 2^−∆∆CT^ analysis of the relative gene expression levels was used for gene expression determination. A list of real-time PCR sequence-specific primers is shown in Table 3.

**Table 3:** Primer sequence

Species	Gene		Primer sequence (5′- 3′)
Human	Collagen I	FORWARD	TGATCGTGGTGAGACTGGTCCTG
		REVERSE	CTTTATGCCTCTGTCGCCCTGTTC
Human	α-SMA	FORWARD	CTTCGTTACTACTGCTGAGCGTGAG
		REVERSE	CCCATCAGGCAACTCGTAACTCTTC
Human	Vimentin	FORWARD	CCTTCGTGAATACCAAGACCTGCTC
		REVERSE	AATCCTGCTCTCCTCGCCTTCC
Human	GAPDH	FORWARD	GGAAGCTTGTCATCAATGGAAATC
		REVERSE	TGATGACCCTTTTGGCTCCC
Human	NR4A1-1	FORWARD	AGGCTCAGGAGAGATCAGGGTGGAA
		REVERSE	GCTCTGACCAGTTATCACCTGCCCG
Human	NR4A1-2	FORWARD	GGGCAGGTGATAACTGGTCAGAGCT
		REVERSE	AGGTGGTGGCACACTGGGTTGGAAC
Human	NR4A1-3	FORWARD	CAGGCTCCACCCGGTTCTGAAATTC
		REVERSE	AACAGCTCTGGCTCCGCTCCACAAG
Human	NR4A1-4	FORWARD	CTCCAGGAAGGGCTTGGGAAGGTGT
		REVERSE	AGAATAACCAGCGGGAGGGCCAGAG

#### Statistical methods

SPSS 26.0 software was used for processing and analysis, and the measurement data was represented by mean ± standard deviation. Normality and homogeneity of variance tests were conducted for each group of data. Tukey test was used for inter-group comparison. *P* < 0.05 indicates that the difference has statistical significance.

## Results

### Knocking down NR4A1 can aggravate renal pathological damage in UUO mice

**Fig. 1:**
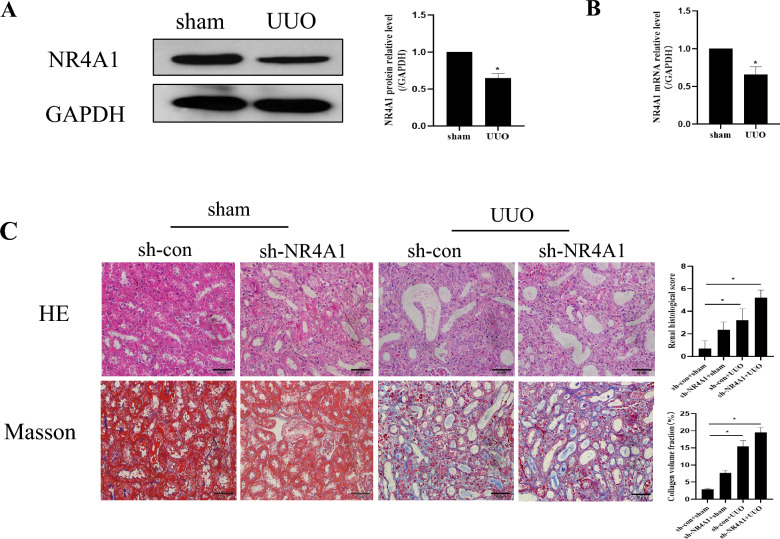
Knocking down NR4A1 can aggravate renal pathological damage in UUO mice. **a** Western blot to examine NR4A1 expression in mice (n = 5). **b** qRT-PCR to examine NR4A1 expression in mice (n = 5). **c** HE and Masson to examine morphological changes and inflammatory cell infiltration in NR4A1 Knocking down mice (n = 6). The data are presented as the mean ± SD, ^*^P < 0.05

Our preliminary sequencing and research results showed that the expression of NR4A1 was decreased in UUO rats (Xu et al. 2023). To further validate the sequencing results, we detected the expression of NR4A1 in UUO mice. The results showed that the mRNA and protein expressions of NR4A1 were decreased in UUO mice (*P* < *0.05*) (Fig. 1a and b).

To further clarify the role of NR4A1 in CKD, we constructed an NR4A1 kidney knockdown UUO mice model. Thereafter, we evaluated renal injury using HE and Masson staining. The HE staining showed that UUO could induce significant infiltration of inflammatory cells and disordered arrangement of renal tubules. Knocking down NR4A1 could exacerbate infiltration of inflammatory cells and more severe kidney damage (*P* < *0.05*) (Fig. 1c). The results of Masson showed that it exhibited collagen deposition in UUO mice, but collagen deposition became more pronounced after knocking down NR4A1 (*P* < *0.05*) (Fig. 1c). The above results indicate that knocking down NR4A1 can aggravate UUO-induced kidney damage.

### Knocking down NR4A1 can exacerbate renal fibrosis in UUO mice

**Fig. 2:**
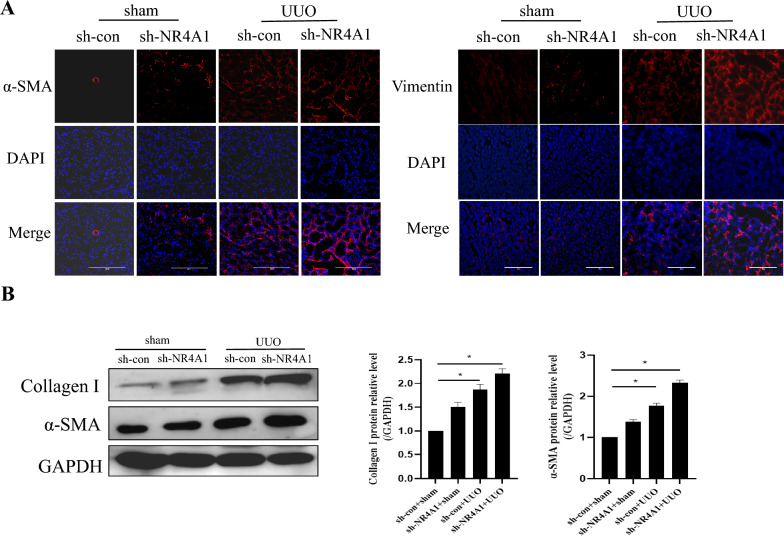
Knocking down NR4A1 can exacerbate renal fibrosis in UUO mice. **a** Immuno- fluorescence to examine α-SMA and Vimentin expression in mice (n = 6). **b** Western blot to examine α-SMA and Collagen I expression in mice (n = 5). The data are presented as the mean ± SD, ^*^P < 0.05

Renal fibrosis is mainly manifested by the deposition of collagen and the proliferation of myofibroblasts. It has been proved that α-smooth muscle actin (α-SMA) and Vimentin are the markers of myofibroblasts (Li et al. 2019). Thus, we detected the expressions of α-SMA, Vimentin and Collagen I to measure the degree of renal fibrosis. The results showed that compared to the sham group, the expressions of α-SMA, Vimentin and Collagen I were significantly increase in kidney tissue of UUO mice. However, UUO mice with NR4A1 knockdown had significantly higher levels of the above proteins compared to the UUO mice without NR4A1 knockdown (*P* < *0.05*) (Fig. 2a and b). These results indicate that knocking down NR4A1 can exacerbate UUO-induced renal fibrosis.

### Cytosporone B can improve UUO-induced renal fibrosis

**Fig. 3:**
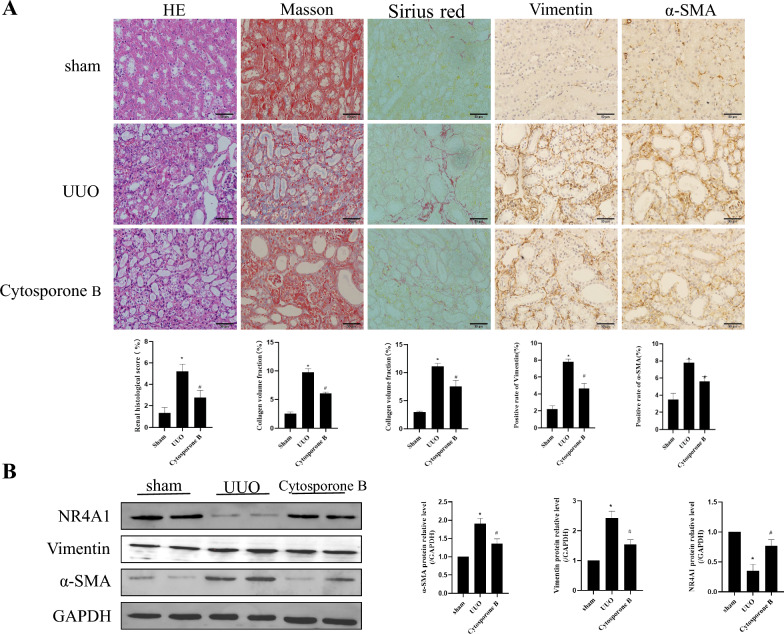
Cytosporone B can improve UUO-induced renal fibrosis. **a** HE, Masson and Sirius red to examine morphological changes and inflammatory cell infiltration in mice (n = 6). Immunoh- istochemical to examine α-SMA and Vimentin expression in mice (n = 6). **b** Western blot to examine NR4A1, Vimentin, and α-SMA expression in mice (n = 5). The data are presented as the mean ± SD, ^*^P < 0.05 versus the sham group. ^#^*P* < 0.05 versus the UUO group

The above results have shown that knocking down NR4A1 can induce renal pathological damage and exacerbate renal fibrosis. To further clarify the role of NR4A1 in renal fibrosis, we intervened UUO mice with the NR4A1 agonist Cytosporone B. HE staining results showed that UUO could induce extensive infiltration of inflammatory cells, resulting in renal tubular atrophy and deformation. However, Cytosporone B could significantly reduce inflammatory cell infiltration and alleviate UUO-induced renal damage (*P* < 0.05) (Fig. 3a). In addition, Masson and Sirius Red staining results showed a significant increase of collagen deposition in UUO mice, while Cytosporone B could significantly alleviate UUO-induced renal collagen deposition (*P* < 0.05) (Fig. 3a). The IHC results showed that the protein expressions of α-SMA and Vimentin were increased significantly in the UUO group compared with the Sham group, but Cytosporone B could significantly decrease these proteins’ expression (*P* < 0.05) (Fig. 3a). Additionally, we used western blot to detect the expression of NR4A1, Vimentin, and α-SMA. We found that the results were consistent with the IHC results (*P* < 0.05) (Fig. 3b). These above results indicate that activating NR4A1 can improve UUO-induced renal fibrosis.

### NR4A1 overexpression can suppress TGF-β1-induced fibrotic effect in HK-2

**Fig. 4:**
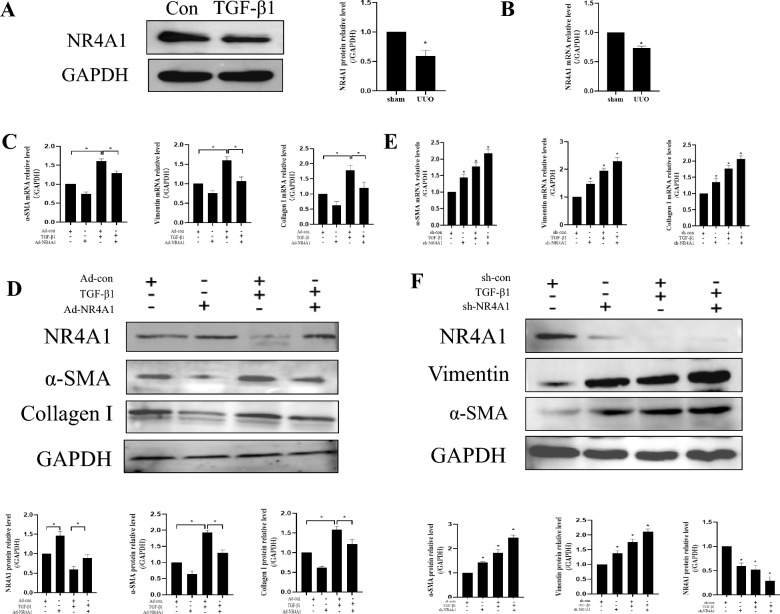
NR4A1 overexpression can suppress TGF-β1-induced fibrotic effect in HK-2. **a** Western blot to examine NR4A1 expression in HK-2 (n = 3).** b** qRT-PCR to examine NR4A1 expression in HK-2 (n = 3).** c** qRT-PCR to examine α-SMA, Vimentin and Collagen I expression in HK-2 (n = 3). **d** Western blot to examine α-SMA and Collagen I expression in HK-2 (n = 3). (**e**) qRT-PCR to examine α-SMA, Vimentin and Collagen I expression in HK-2 (n = 3). (**f**)Western blot to examine Vimentin and α-SMA expression in HK-2 (n = 3). The data are presented as the mean ± SD, ^*^P < 0.05

We also determined the role of NR4A1 in a cell model of renal fibrosis that is induced by TGF-β1. The results showed that the expression of NR4A1 was significantly reduced in HK-2 treated with TGF-β1 (*P* < *0.05*) (Fig. 4a and b). We further specifically knocked down or overexpressed NR4A1 in HK-2 cells with or without TGF-β1 to further confirm its role in renal fibrosis. The NR4A1 overexpression could inhibit TGF-β1-induced fibrotic effect by decreasing the expressions of α-SMA and Collagen I in HK-2 (*P* < *0.05*) (Fig. 4c and d). However, NR4A1 knockdown could exacerbate TGF-β1-induced fibrotic effect by increasing their expressions in HK-2 (*P* < *0.05*) (Fig. 4e and f). Taken together, these results indicate that NR4A1 is downregulated in the cell model of renal fibrosis that is induced by TGF-β1 treatment, and NR4A1 overexpression can inhibit renal fibrosis.

### Knocking down SPDEF can exacerbate renal pathological damage in UUO mice

**Fig. 5:**
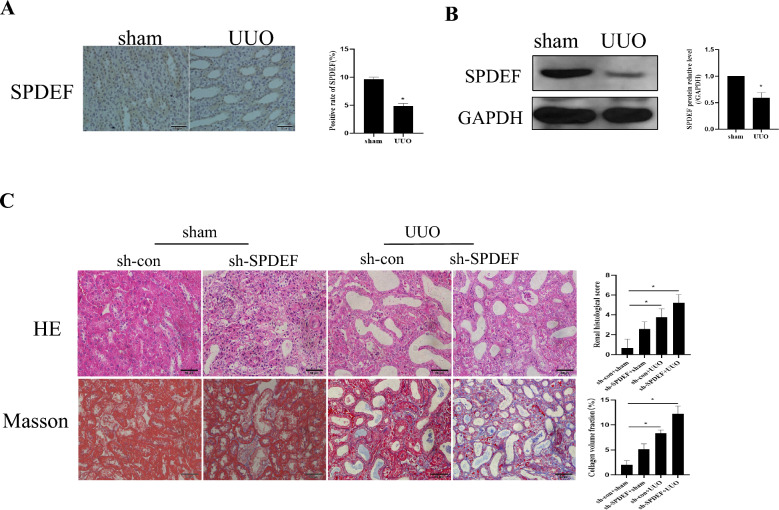
Knocking down SPDEF can exacerbate renal pathological damage in UUO mice. **a** Immunohistochemical to examine SPDEF expression in mice (n = 6).** b** Western blot to examine SPDEF expression in mice (n = 5).** c** HE and Masson to examine morphological changes and inflammatory cell infiltration (n = 6). The data are presented as the mean ± SD,^*^P < 0.05

The above results clarify that it may antagonize renal fibrosis if NR4A1 expression can be up-regulated. However, its exact mechanism is still unclear. Previous studies have shown that SPDEF can activate the expression of NR4A1 (Wang et al. 2021). Therefore, we first detected the expression of SPDEF in renal fibrosis. We found that the expression of SPDEF was significantly reduced in the kidney tissue of UUO mice compared to the sham group (*P* < *0.05*) (Fig. 5a and b).

To further demonstrate the role of SPDEF in renal fibrosis, we construct an SPDEF knockdown UUO mice model by injecting a virus into the renal cortex. After knocking it down, we detected renal pathological damage by HE and Masson staining. The HE results showed that the renal tubules in the sham group were arranged regularly and neatly, with a small amount of inflammatory cell infiltration. Compared with the sham group, there is a significant increase in inflammatory cell infiltration and irregular arrangement of renal tubules in UUO mice. Nevertheless, knocking down SPDEF could aggravate UUO-induced renal damage (*P* < 0.05) (Fig. 5c). Besides, the Masson staining results showed that the UUO group showed more significant collagen deposition than the sham group, while SPDEF knockdown could increase UUO-induced collagen deposition (*P* < 0.05) (Fig. 5c). The above results demonstrate that SPDEF knockdown can promote UUO-induced kidney damage.

### Knocking down SPDEF can exacerbate renal fibrosis in UUO mice

**Fig. 6:**
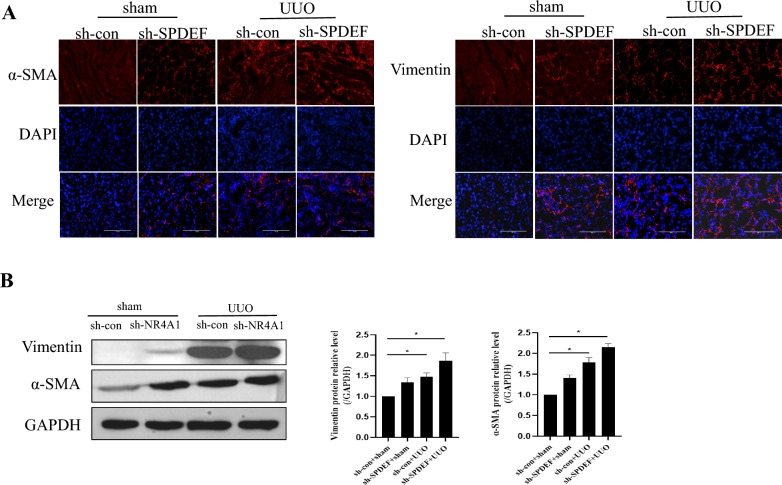
Knocking down SPDEF can exacerbate renal fibrosis in UUO mice. **a** Immunofl- uorescence to examine α-SMA and Vimentin expression in mice (n = 6).** b** Western blot to examine α-SMA and Vimentin expression in mice (n = 5). The data are presented as the mean ± SD, ^*^P < 0.05

To further validate the effect of SPDEF on renal fibrosis, we tested the expression of renal fibrosis related indicators α-SMA and Vimentin. The results showed that compared to the sham group, the expression of α-SMA and Vimentin significantly increased in UUO group. However, knocking down SPDEF could enhance UUO-induced the expression of α-SMA and Vimentin (*P* < 0.05) (Fig. 6a and b). The above results indicate that SPDEF knockdown can exacerbate UUO-induced renal fibrosis.

### SPDEF overexpression can inhibit TGF-β1-induced fibrotic effect in HK-2

**Fig. 7:**
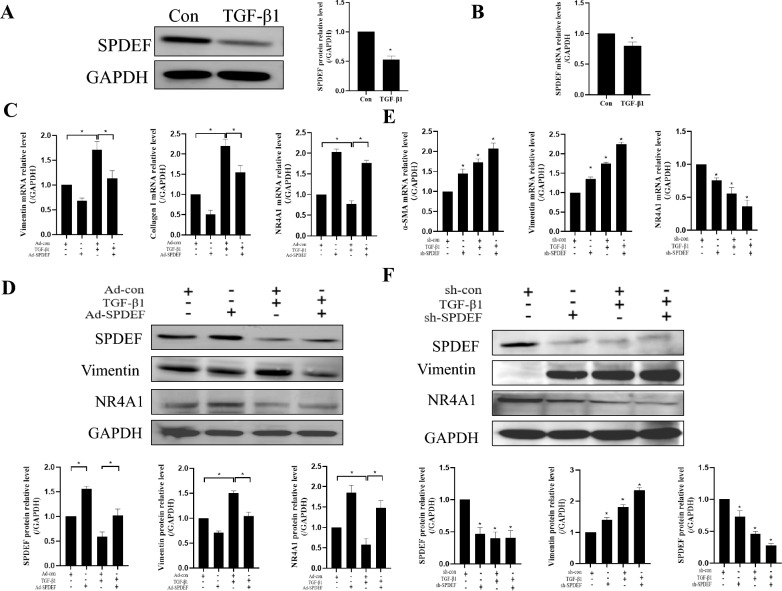
SPDEF overexpression can suppress TGF-β1-induced fibrotic effect in HK-2. **a** Western blot to examine SPDEF expression in HK-2 (n = 3). **b** qRT-PCR to examine SPDEF expression in HK-2 (n = 3). **c** qRT-PCR to examine NR4A1, Vimentin and Collagen I expression in HK-2 (n = 3). **d** Western blot to examine SPDEF, Vimentin and NR4A1 expression in HK-2 (n = 3). **e** qRT-PCR to examine α-SMA, Vimentin and NR4A1 expression in HK-2 (n = 3). **f** Western blot to examine Vimentin, SPDEF and NR4A1 expression in HK-2 (n = 3). The data are presented as the mean ± SD, ^*^P < 0.05

To further demonstrate the role of SPDEF in renal fibrosis, we determined the role of SPDEF in a cell model of renal fibrosis that was induced by TGF-β1. The results showed that the expression of SPDEF was significantly reduced in HK-2 treated with TGF-β1 (*P* < *0.05*) (Fig. 7a and b). We also utilized virus transfection cells to specific knock down or over express SPDEF. The results show that knocking down SPDEF can significantly exacerbate TGF-β1-induced fibrosis (*P* < 0.05) (Fig. 7e and f), while over expression of SPDEF can alleviate TGF-β1-induced fibrosis (*P* < 0.05) (Fig. 7c and d). These results indicate that SPDEF overexpression can improve TGF-β1-induced fibrosis.

### SPDEF directly activates NR4A1 transcription in HK-2 cells

**Fig. 8:**
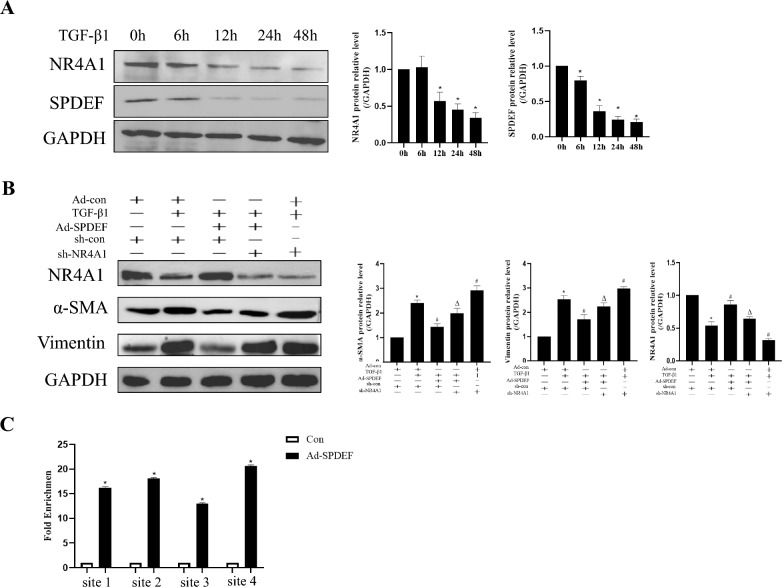
SPDEF directly activates NR4A1 transcription in HK-2. **a** Western blot to examine NR4A1and SPDEF in HK-2 (n = 3). **b** Western blot to examine NR4A1, α-SMA and Vimentin expression in HK-2 (n = 3). **c** ChIP-qPCR to examine the relationship between SPDEF and NR4A1 (n = 3). The data are presented as the mean ± SD,^*^P < 0.05 versus the Con group. ^#^*P* < 0.05 versus the TGF-β1 group. ^∆^*P* < 0.05 versus the Ad-SPDEF + TGF-β1 group

The above results have shown that the expressions of SPDEF and NR4A1 is reduced in animal models of renal fibrosis. However, it has been proved that SPDEF can transcriptionally activate NR4A1 (Wang et al. 2021). Therefore, we speculate that SPDEF can inhibit renal fibrosis by transcriptionally activating NR4A1. To understand the timing and dynamics of SPDEF and NR4A1 expression, we first observed whether their expressions would be affected by TGF-β1 with the duration of its stimulation in HK2 cells. The results showed a transient rise in NR4A1 expression at 6 h followed by a progressive decrease, while the expression of SPDEF consistently showed a decreasing trend (Fig. 8a). This result suggests that there may be a possible interaction between NR4A1 and SPDEF (*P* < *0.05*). To further explore thire relationship, we transfected the cells with a virus that specifically overexpresses SPDEF and simultaneously transfected with a virus of sh-NR4A1. The results showed that the expression of NR4A1 was significantly reduced when SPDEF was knocked down, but NR4A1 could up-regulate with overexpression of SPDEF. Overexpression of SPDEF significantly inhibited TGF-β1-induced expression of renal fibrosis-related genes, but the above effects were significantly suppressed when NR4A1 was also knocked down (*P* < *0.05*) (Fig. 8b).

To further validate the relationship between SPDEF and NR4A1 regulation, we transferred HK-2 cells with a virus to overexpress SPDEF. It has been proved that SPDEF can bind to four candidate sites of NR4A1 promoter, thereby affecting its transcriptionally (Wang et al. 2021). Thus, we performed ChIP-qPCR to verify that. Our results demonstrated that the quantity of SPDEF-bound sites in the NR4A1 promoter was significantly higher in Ad-SPDEF group (*P* < *0.05*) (Fig. 8c). The results clarify that SPDEF can inhibit renal fibrosis by transcriptionally activating NR4A1.

## Discussion

CKD has emerged as a significant public health issue in recent years, with a rising number of case every year. At present, the treatment of CKD is based on symptomatic therapy, which may provide good clinical results in the short-term, but it has limited effectiveness over the long term. Additionally, this treatment method can be quite expensive and places a considerable financial burden on individuals and society as a whole (Luyckx et al. 2020). Renal fibrosis, which includes glomerulosclerosis and renal interstitial fibrosis, is a common pathway that leads to the progression of various CKD to end-stage renal failure (Lai et al. 2020; Stewart et al. 2018). If we can further clarify the mechanism behind the occurrence and development of renal fibrosis, it would have significant clinical implications for treating CKD. Previous studies have established that oxidative damage, inflammatory response, phenotypic transformation are all closely related to renal fibrosis (Wu et al. 2022; Liu et al. 2018). However, its exact regulatory mechanism that governs renal fibrosis is still unclear.

As a transcription factor, NR4A1 belongs to the nuclear receptor superfamily and can be involved in cell proliferation, apoptosis, endocrine and immune diseases through a variety of pathways, and plays an important role in the development of a variety of diseases. It plays an important role in fibrotic diseases (Westbrook et al. 2014). But its role is controversial in fibrotic diseases. Some studies showed that it can promote the progression of fibrosis. For example, NR4A1 can exacerbate cardiac fibrosis by promoting endothelial mesenchymal transition (Chen et al. 2021). In addition, it can also promote the development of fibrosis through multiple pathways in various models of hepatic and ovarian fibrosis (Hiwatashi et al. 2017; Fuchs et al. 2023; Cui et al. 2023; Huang et al. 2022). However, other studies proved that NR4A1 was also an endogenous inhibitor of fibrosis. NR4A1 can significantly inhibit fibroblast activation to repress intestinal fibrosis during the development of inflammatory bowel disease and vocal fold fibrosis (Pulakazhi Venu et al. 2021; Hiwatashi et al. 2018). As an endogenous inhibitor of TGF-β1, NR4A1 deficiency can exacerbate renal injury (Palumbo-Zerr et al. 2015). These studies indicate that NR4A1 can play an important role in the development of CKD. Our previous findings showed that NR4A1 expression was reduced in the UUO mice (Xu et al. 2023). We also proved that it cloud regulate renal fibrosis probably by affecting mitochondrial function and angiogenesis (Wang et al. 2024; He et al. 2022). Here, we again specifically knocked down NR4A1 in the mice kidney. We found that knocking down NR4A1 could aggravate UUO-induced renal fibrosis. This result was consistent with our previous study. The above conclusions show that NR4A1 is crucial in CKD, and if the specific mechanism of NR4A1 down regulation can be explained clearly, it will be crucial for clinical and basic research.

The ETS (E26 transformation-specific) family belongs to the nuclear effectors of signal transduction pathways. All members of the family have a highly conserved DNA-binding structural domain, the ETS structural domain, which binds to DNA sites containing GGA (A/T) mats to initiate transcription of the corresponding genes and realize a variety of biological functions (Li et al. 2023). SPDEF is the only transcription factor in the ES family that binds preferentially to GGAT sequence, whereas all other members bind preferentially to this sequence (Marko et al. 2013). Previous studies have shown that SPDEF expression is reduced in animal models of pulmonary fibrosis (Plantier et al. 2011). SPDEF can negatively regulate the development of fibrosis. But the role of SPDEF in renal fibrosis is unclear. Therefore, we examined the expression of SPDEF in UUO mice or HK-2 cells treated with TGF-β1, and the results showed a significant decrease in the expression of SPDEF in both UUO mice and HK-2 cells treated with TGF-β1. Additionally, we found that knocking down SPDEF could aggravate UUO-induced or TGF-β1-induced renal fibrosis. Our results have demonstrated that both SPDEF and NR4A1 can negatively regulate the development of renal fibrosis. Previous studies have shown that SPDEF can transcriptionally activate NR4A1 (Wang et al. 2021). Our results showed that knockdown of SPDEF significantly inhibited the expression of NR4A1, but when overexpression of SPDEF was performed, the expression of NR4A1 was significantly elevated. Consequently, our ChIP-qPCR results showed that SPDEF could also transcriptionally activate NR4A1.

## Conclusions

NR4A1 is a crucial endogenous inhibitor of TGF-β1, which plays a significant role in the development of CKD. Knockdown of NR4A1 can significantly aggravate UUO-induced renal fibrosis to a great extent. SPDEF is a transcription factor that can activate NR4A1 transcriptionally and subsequently contribute positively in the regulation of renal fibrosis.

## Data Availability

The datasets used and/or analyzed during the current study are available from the corresponding author on reasonable request.
